# 1025. Prediction of Intravenous Immunoglobulin Resistance and Coronary Artery Dilatation in Kawasaki Disease: a Multicenter Study from Oman

**DOI:** 10.1093/ofid/ofab466.1219

**Published:** 2021-12-04

**Authors:** Fatma Al Mwaiti, Zaid Alhinai, Safiya AlAbrawi, Asmhan A L mamari, Reem Abdwani, Khalfan Al Senidi

**Affiliations:** 1 Omsb, Masqat, Masqat, Oman; 2 Sultan Qaboos University, Muscat, Masqat, Oman; 3 MOH, Masqat, Masqat, Oman; 4 SQU, Masqat, Masqat, Oman; 5 SQUH, Masqat, Masqat, Oman

## Abstract

**Background:**

Prediction of intravenous immunoglobulin (IVIG) resistance and coronary artery dilatation continues to be a challenge in the management of Kawasaki disease. Significant differences exist among different populations.

**Methods:**

Children < 13 years of age who presented to the two main tertiary care hospitals in Oman (Royal Hospital and Sultan Qaboos University Hospital) between 2008 and 2019 with a diagnosis of Kawasaki disease were included. Diagnosis was confirmed and clinical, laboratory and echocardiography data was systematically collected and checked for accuracy. The primary outcome was the presence of IVIG resistance or coronary artery dilatation at the 6-week follow-up. Bivariate analysis was used to identify significant predictors of the primary outcome, followed by multivariable logistic regression to determine independent predictors. The Muscat Index of Kawasaki disease Severity (MIKS) score was created based on the results.

**Results:**

156 children with Kawasaki disease were included. Median age was 2.1 years (IQR 0.9-3.8), and 64% were males. All patients received IVIG, 26 (17%) received steroids, and one received infliximab. Coronary dilatation was identified in 41 (26%) patients on initial echocardiogram, and 26 (18%) at the 6-week follow-up visit. Variables significantly associated with the primary outcome were age ≤15 months (P=0.031), hemoglobin (P=0.009), WBC count (P=0.002), absolute neutrophil count (P=0.006), and CRP ≥150 mg/L (P=0.015). These variables in addition male gender (P=0.058), ALT >80 IU/L (P=0.10) and serum sodium (P=0.10), were entered into multivariable logistic regression. A predictive model based on CRP ≥150 mg/L (LR=2.2, P=0.049), male gender (LR=2.1, P=0.095) and WBC (LR=1.1, P=0.017) resulted, and it was used as basis for the MIKS score (Table 1). The MIKS score performed favorably to the Kobayashi score in its sensitivity to predict the primary outcome and its separate components (Table 2). Combining the MIKS score with other high-risk criteria had a sensitivity of 95% in predicting the primary outcome and a specificity of 56%.

Table 1. Calculation of the Muscat Index of Kawasaki disease Severity (MIKS) score

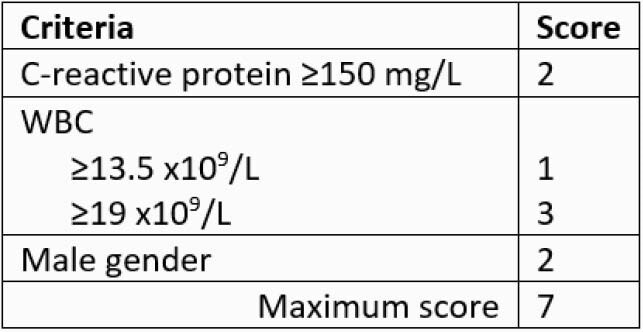

Table 2. Sensitivity, specificity and P value for the Kobayashi, MIKS, and combined high risk criteria in predicting IVIG resistance, coronary dilatation at 6 weeks, separately or in combination, among patients with Kawasaki disease. MIKS: Muscat Index of Kawasaki disease Severity. *High risk: presence of coronary artery dilatation on initial echocardiogram or age <1>

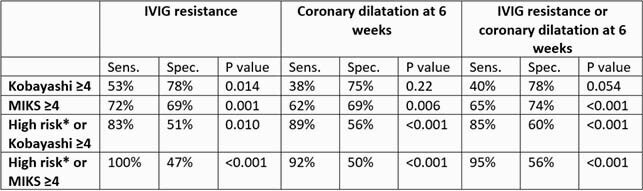

**Conclusion:**

The MIKS score predicts IVIG resistance and coronary artery dilatation in Kawasaki disease in our setting, with favorable performance compared to the Kobayashi score.

**Disclosures:**

**All Authors**: No reported disclosures

